# Supplementary material for: Association between lower limb alignment and low back pain: A systematic review with meta-analysis
Source: PLoS One. 2024 Oct 10;19(10):e0311480. doi: 10.1371/journal.pone.0311480 (PMC11466400; doi:10.1371/journal.pone.0311480)
Supplement: Attachment — Submitted filename: Response to reviewers.docx [file pone.0311480.s004.docx]

**Dear editor and reviewers;**

We thank you for your insightful and constructive suggestions. Based on your helpful suggestions, we were able to further improve our manuscript. We carefully considered and addressed all your specific comments and revised the text if necessary.

Please find your comments in bold and our responses in italic font. In the manuscript, the highlighted parts in green are the revisions made based on the comments of reviewer1 and the highlighted parts in yellow are the revisions made based on the comments of reviewer2.

**In the online submission form, you indicated that [The data underlying the results presented in the study are available from (**[Saeideh.abbasi@ut.ac.ir](mailto:Saeideh.abbasi@ut.ac.ir)**).].
All PLOS journals now require all data underlying the findings described in their manuscript to be freely available to other researchers, either 1. In a public repository, 2. Within the manuscript itself, or 3. Uploaded as supplementary information.**

*Thank you for your comment. Edited as bellow:*

*All data are presented in this study.*

**Reviewer #1**

**Abstract**

**1* Keywords. Please add LBP to Keywords.**

*Thank you for your comment. Edited as bellow:*

***Keywords:*** *low back pain, postural alignment, lower extremity, biomechanics, knee, ankle, hip*

**Methods**

**2* Please update search strategies.**

*Thank you for your notice.*

*It had already been updated to May 2024 in the Abstract but not in the Methods section. Now it is corrected as bellow in the Methods section too:*

*A comprehensive search was conducted in PubMed, Scopus, Web of Science, and Embase, from database inception to May 2024.*

**Reviewer #2**

*Thank you for taking the time to provide detailed feedback on our manuscript. We appreciate your insights and constructive criticism. Your comments will be invaluable in refining our study. We carefully address each of the points you have raised to enhance the clarity of our rationale, align the benefits with the actual outcomes, improve the accuracy of our results, and enhance the structure of our discussion. Your input will undoubtedly contribute to the overall quality of our work.*

**Title**

**1* Please revise title so that it more accurately reflects the review. Avoid titles written as questions. Reformat – do not make as a question**

*Thank you for your comment. Edited as bellow:*

***Association between lower limb alignment and low back pain: A systematic review with meta-analysis***

**2* The manuscript needs a structured abstract with distinct headings.**

*This section is written based on the PLOS ONE style templates:* [*https://journals.plos.org/plosone/s/file?id=wjVg/PLOSOne_formatting_sample_main_body.pdf*](https://journals.plos.org/plosone/s/file?id=wjVg/PLOSOne_formatting_sample_main_body.pdf)*.*

*But if it is necessary we will change the format.*

**Introduction**

**3* Page 1, lines 34-35: " ALBP refers to pain persisting for less than six weeks, whereas CLBP is defined by pain lasting for 12 weeks or longer." The sentences need valid citation. Please add.**

*Thank you for your comment. We added a research paper to the reference:*

*ALBP refers to pain persisting for less than six weeks, whereas CLBP is defined by pain lasting for 12 weeks or longer (5).*

**4*** **Page 1, lines 39-40: " LBP is caused by poor posture, muscle imbalances, and altered lower limb alignment, influenced by the complex interplay of pelvis, hip, knee, and ankle joints.” According to previous studies there is no causal relationship between these factors and LBP. Please revise.**

*Thank you for your comment. We clarified as bellow:*

*Since the kinetic chain concept highlights how dysfunctions in the lower limbs, such as poor posture, muscle imbalances, and altered lower limb alignment, can affect pelvic positioning, spinal posture, and upper body mechanics, this cascading effect can lead to musculoskeletal disorders.[8].*

**5*** **Because the authors excluded “treatment studies” second objectives of the study must be stated clearly. Please revise.**

*Thank you for your comment. The following sentence was removed since it was not in the scope of our research.*

*“Thereby enhancing the understanding of movement impairments helps to develop effective treatment strategies.”*

**Methods**

**6*** **How the alignment as a variable was defined? In what conditions? Dynamic or static? define all the methods used to decide which objective results for alignment to collect.**

*Thank you for your comment. We clarified as bellow:*

*In this study, alignment refers to the positioning and orientation of body segments in relation to a defined reference line or anatomical landmarks that were measured under dynamic or static conditions.*

*Also, we have added a ‘Tool/ condition’ column to the relevant Table2, specifying which alignments were measured and under which conditions, whether dynamic or static. These revisions aim to provide a clearer understanding of the alignment variable used in the studies reviewed.*

**7*** **I would like to see more information about the methods used to assess risk of bias for different studies included in this review. What method used for appraising different studies?**

*Thank you for your comment. We clarified and edited as bellow:*

*Two authors (S.A. and S.H.M.) independently assessed the methodological quality of the included studies using the modified Downs and Black checklist. This checklist consists of 27 questions relating to quality of reporting (ten questions), external validity (three questions), internal validity (bias and confounding) (13 questions), and statistical power (one question) (S1. Table). Fourteen of them that are related to our study, marked yes (=1) or no (=0). When there was uncertainty or disagreement between the initial reviewers on how to score a particular question, the matter was referred to a third reviewer (F.KH) for discussion (yes (=2) or partially (=1) and no (=0). Percentage agreement reliability between two reviewers is shown in Table 3. The overall quality of evidence was classified into four levels: high, moderate, and low [22].*

**8*** **Page 3, lines 96-97: “Two authors…independently assessed the methodological quality of the included studies using the modified Downs and Black checklist with high reliability (0.89) and validity (0.90).” % agreements and kappa values are usually reported for raters in review studies. It is not clear why validity index was reported in this review. Please comment.**

*Thank you for your comment. Since reliability and validity of this scales aren’t usually reported, we removed it and clarified as bellow:*

*Two authors (S.A. and S.H.M.) independently assessed the methodological quality of the included studies using the modified Downs and Black checklist. This checklist consists of 27 questions relating to quality of reporting (ten questions), external validity (three questions), internal validity (bias and confounding) (13 questions), and statistical power (one question) (S1. Table). Fourteen of them that are related to our study, marked yes (=1) or no (=0). When there was uncertainty or disagreement between the initial reviewers on how to score a particular question, the matter was referred to a third reviewer (F.KH) for discussion (yes (=2) or partially (=1) and no (=0). Percentage agreement reliability between two reviewers is shown in Table 3. The overall quality of evidence was classified into four levels: high, moderate, and low [22].*

**Result**

**9*** **Page 8, lines 164-165: “Two studies assessed knee alignment during walking, and sex specific kinematic data were reported in some studies.” The sentence needs valid citation. Please add.**

*Thank you for your comment. We added two references related to the sentence.*

*Two studies assessed knee alignment during walking, and sex-specific kinematic data were reported in some studies (26,36)****.***

**Discussion**

**10*** **The results for alignment in dynamic and static conditions were presented. It would be good to “synthesize and analyze” the role of these two conditions and LBP separately. Can they further comment on this?**

*Thank you for your comment. Static and dynamic conditions were separated in the results for meta-analysis. As requested, we discussed them separately in the discussion.*

**11*** **Page 11, lines 214-215: “…possibly due to increased soft tissue elasticity in females.” The sentence needs valid citation. Please add.**

*Thank you for your comment. We edited as bellow:*

*possibly due to the fact that although females have more joint mobility and ligament laxity in the foot compared to males, which leads to a more dynamic internal longitudinal arch. But females rely more on the toe flexor muscles to support the arch, which may explain the difference in arch stability between the sexes (43–45)*

**12*** **The section for limitation must be revised. Discuss the factors that limit the generalizability of study results.**

*Thank you for your comment. We clarified as bellow:*

*The reliance on various static measurement indexes in the included studies significantly restricts the capacity to reach definitive conclusions and apply findings across different clinical contexts. A consensus must be reached on the use of a standardized static measurement index to improve comparability and generalizability. Moreover, neglecting to account for sex and pain duration in the data analysis of some included studies overlooks significant differences in treatment responses, potentially invalidating conclusions for various populations. Researchers should consistently incorporate sex differences and pain duration categories to improve insights and relevance.*

*Inconsistent measurement methods and terminology (e.g., pes planus, pronation, ND, ankle ROM) create ambiguity that complicates result comparisons across studies, undermining the reliability of evidence. Standardizing biomechanical factors and terminology is crucial for enhancing clarity and facilitating reliable comparisons.*

**13*** **Page 12, lines 285-287: “Considering the complex and multifactorial nature of the relationship between lower extremity alignment and LBP, clinicians should take alignments and targeted interventions into account in the management of LBP.” Based on the results of this review there were no causal relationship between alignment and LBP. Please revise.**

*Thank you for your comment. We clarified as bellow:*

*Considering the complex and multifactorial nature of the association between lower extremity (such as ankle and hip) alignments and LBP, clinicians should still consider lower extremity alignment as one of important factors in the management of LBP.*

**14* The implications of the findings of the study for future research should also be clearly stated.**

*Thank you for your comment. We edited as bellow:*

*Future research should explore the role of lower extremity alignment in managing low back pain, optimize interventions, and investigate longitudinal studies for sustained improvements. Additionally, it should consider assessing dynamic alignment alongside static alignment for personalized treatment strategies.*
