# Supplementary material for: Association between lower limb alignment and low back pain: A systematic review with meta-analysis
Source: PLoS One. 2024 Oct 10;19(10):e0311480. doi: 10.1371/journal.pone.0311480 (PMC11466400; doi:10.1371/journal.pone.0311480)
Supplement: Attachment — Submitted filename: Response to reviewers.docx [file pone.0311480.s005.docx]

**Dear editor and reviewers;**

We thank you for your insightful and constructive suggestions. Based on your helpful suggestions, we were able to further improve our manuscript. We carefully considered and addressed all your specific comments and revised the text if necessary.

Please find your comments in bold and our responses in italic font.

**Journal Requirements:

1* Please review your reference list to ensure that it is complete and correct. If you have cited papers that have been retracted, please include the rationale for doing so in the manuscript text, or remove these references and replace them with relevant current references. Any changes to the reference list should be mentioned in the rebuttal letter that accompanies your revised manuscript. If you need to cite a retracted article, indicate the article’s retracted status in the References list and also include a citation and full reference for the retraction notice.**

*Thank you for bringing this important issue to our attention. We have thoroughly  reviewed all our references, considering the significance of the point you raised We confirm that none of refrences have been retracted. Therefore, there is no need to change the references or provide an explanation regarding the use of retracted references in the manuscript.*

**Reviewer #1**

**Methods**

**1* The authors updated search strategy. But they did not provide the information about the data obtained in the result section. Please add it.**

*Thank you for your comment.*

*We have updated the search strategy through September 2024, and the flowchart has been revised accordingly. based on the screening, no papers were added to our review.*
